# Congenital cytomegalovirus and associations with maternal HIV serostatus and child health outcomes in Uganda

**DOI:** 10.1186/s12879-026-13655-2

**Published:** 2026-05-28

**Authors:** Julian Adong, Nitin Arora, Elias Kumbakumba, Adeline A. Boatin, Anacret Byamukama, Ingrid V. Bassett, Stephen Asiimwe, Lynn T. Matthews, Suresh Boppana, Joseph Ngonzi, Lisa M. Bebell

**Affiliations:** 1 Department of Paediatrics and Child Health, Mbarara University of Science and Technology, Mbarara, Uganda; 2 University of Alabama at Birmingham, Heersink School of Medicine, Birmingham, USA; 3 Department of Obstetrics and Gynecology, Massachusetts General Hospital, Boston, USA; 4 Harvard Medical School, Boston, MA USA; 5 Kabwohe Clinical Research Centre, Kabwohe, Uganda; 6 Center for Global Health, Massachusetts General Hospital, Boston, USA; 7 Department of Obstetrics and Gynecology, Mbarara University of Science and Technology, Mbarara, Uganda; 8 Medical Practice Evaluation Center, Division of Infectious Diseases, Department of Medicine, Massachusetts General Hospital, Boston, USA; 9 Yale University School of Medicine, New Haven, Connecticut, USA; 10 mbarara, Uganda

**Keywords:** CMV, HIV-exposed infant, Hearing screen, Sub-Saharan Africa

## Abstract

**Introduction:**

Cytomegalovirus (CMV) is the most common congenital infection globally, and the leading cause of non-genetic hearing loss and neurodevelopmental disability. The burden is highest in low- and middle-income countries where HIV is also highly prevalent. Women living with HIV have a higher prevalence of CMV and are more likely to transmit CMV to their infants. Despite this, congenital CMV (cCMV) and newborn hearing screening are not routinely available in sub-Saharan Africa (sSA) resulting in missed diagnoses and delayed treatment. Additionally, data comparing cCMV prevalence in HIV-exposed and non-exposed infants is limited. We aimed to determine cCMV prevalence among HIV-exposed and -unexposed neonates in Uganda, describe associated clinical characteristics, and assess infant hearing outcomes.

**Methods:**

This study was nested within a prospective cohort of pregnant women with and without HIV, followed with their infants through early childhood. Eligible participants were 18 years or older, pregnant, and planned to deliver at one of two clinics. We tested newborn saliva for CMV using a real-time PCR. Hearing screening was performed using a pre-approved screening algorithm. Maternal and neonatal medical characteristics and diagnoses were abstracted from clinical records.

**Results:**

A total of 793 women and their newborns were enrolled. Mean maternal age was 28 (standard deviation [SD] 6) years, and parity was 3 (SD, 2.0). The mean birth weight was 3.0 (SD, 0.5) kilograms and gestational age was 37 (SD, 2.0) weeks. Overall, 677 out of 793 (84.7%) neonatal saliva samples were tested for cCMV, 23 out of 677 (3.4%) were positive with no difference by maternal HIV status. cCMV was not associated with failed hearing screen. However, lower one-minute Apgar and jaundice were associated with cCMV.

**Conclusion:**

cCMV prevalence was high in this Ugandan cohort and did not differ by HIV exposure. Hearing outcomes were similar by cCMV status. Future studies should include a comprehensive clinical and audiological assessment for both HIV-exposed and -unexposed neonates, and longitudinal follow-up to detect hearing loss and other cCMV-associated sequelae.

**Supplementary Information:**

The online version contains supplementary material available at 10.1186/s12879-026-13655-2.

## Introduction

Cytomegalovirus (CMV) infection is widespread globally and nearly all adults in low- and middle-income countries (LMICs) are CMV seropositive by age 40 years [1]. Congenital CMV (cCMV), resulting from intrauterine transmission during primary or non-primary maternal infection is the most common congenital infection worldwide. Although data from sub-Saharan Africa (sSA) are limited, cCMV prevalence is estimated at about 0.4–5% among infants [2–4]. A definitive diagnosis of cCMV requires testing within the first three weeks after birth to distinguish from postnatally acquired CMV.

The burden of cCMV may be higher in sSA due to high HIV prevalence and a synergistic relationship between CMV and HIV infections. Women living with HIV are more likely to have CMV and therefore transmit CMV to their child than women without HIV [5]. Additionally, maternal HIV infection may increase the likelihood of cCMV. Reported cCMV prevalence is higher among HIV-exposed but HIV-uninfected infants (approximately 11.6%) than HIV-unexposed infants (< 5%) [6], but lower than CMV prevalence in infants with HIV, likely reflecting higher CMV viremia in women with HIV [5].

cCMV infection is often asymptomatic in at birth and therefore, many infected infants are not diagnosed at birth [7, 8]. Furthermore, complications of cCMV can appear years after birth [9] making it challenging to attribute specific problems to cCMV. The most common manifestation of cCMV is non-genetic congenital sensorineural hearing loss (SNHL), accounting for approximately 25% of all childhood SNHL cases globally and making cCMV the most common cause of SNHL worldwide [7]. Additionally, cCMV has been associated with long-term cognitive and developmental impairments [10] and mortality through age 5 years [11]. Despite this burden of disease, in sSA, routine cCMV screening and newborn hearing screening are not routinely carried out, resulting in missed diagnoses and delayed intervention. In addition, there are limited comparative data on cCMV burden among HIV-exposed and unexposed infants in this setting.

Given the high co-prevalence of HIV and CMV and lack of routine screening, there is need for sSA-specific data to guide detection and early intervention. We therefore aimed to determine the prevalence of cCMV among HIV-exposed and -unexposed neonates in Uganda, identify clinical characteristics associated with cCMV infection, and assess early hearing outcomes among affected infants. Our goal was to bridge a gap in the literature on cCMV prevalence and symptoms, since timely screening and intervention can improve outcomes, even in low-resource settings [12].

## Methods

### Participant enrollment and data collection

This was pre-specified nested sub-study within a larger prospective parent cohort study, the Placenta, Antibodies and Child Outcomes (PACO) study. PACO enrolled pregnant women with and without HIV and followed mother-baby dyads through early childhood. Women aged 18 years or older were eligible for enrollment if they were pregnant and planned to deliver at Mbarara Regional Referral Hospital (MRRH) or Kabwohe Clinical Research Center (KCRC). Exclusion criteria included multiple pregnancy and HIV-positive serostatus but not taking combination antiretroviral therapy in the last 30 days. At delivery, neonatal weight was measured. Neonatal medical characteristics and diagnoses (presence of jaundice, lethargy, fever, convulsions, difficulty in breathing, poor feeding or required a birth admission) were abstracted from participant files, and enrolled women underwent a standardized interview to collect demographic and health information (supplemental file 1). For women with HIV, details of HIV-specific care were collected from the clinic where they were treated for HIV.

### Saliva collection for CMV testing

We chose saliva for cCMV screening because it is simple to collect, highly sensitive, and PCR results are unlikely to be significantly influenced by breastfeeding or other perinatal exposures [13]. Saliva samples were collected within 48 h after birth and at least 30 min after breastfeeding. Saliva was collected from the neonate using a sterile polyester-tipped applicator. The sterile swab was placed in the neonate’s mouth under the edge of the tongue until it was completely saturated with saliva (approximately 20 s) and then placed into a cryovial and allowed to air-dry for at least 4 h at room temperature before being transferred to the on-site − 80 °C freezer for storage.

### CMV testing

This was conducted at the Mbarara University of Science and Technology research laboratory. Saliva samples were processed and real-time PCR to detect CMV DNA was performed using published methods [14]. Briefly, saliva swabs were thawed and resuspended in PCR-grade water. Real-time PCR was used to measure cycle threshold. All samples were run in duplicate, along with positive and negative (no-template) controls on each panel, using the CMV IE2 exon 5 probe and lyophilized IE2 primers (Applied Biosystems, Massachusetts, USA). A cut-off cycle threshold of < 36.5 was considered positive. To ensure testing accuracy, the eluant from 30 swabs (5% of all samples) were tested at a local reference laboratory (MBN, Mbarara, Uganda) using similar real-time PCR methods to measure cycle threshold. Although saliva samples were collected from all neonates, only the first 677/793 (84.7%) were tested for CMV due to funding constraints.

### Hearing screening and follow-up

Neonates underwent hearing screening 24–72 h after vaginal birth and 48–72 after caesarean birth using a PATH™ Sentiero OAE/Tympanometer/ABR machine, Germering, Germany. Testing was conducted in a quiet room by two research assistants trained in hearing assessment by an Ear, Nose, and Throat clinical officer. Tympanometry was performed first, followed by transitory evoked otoacoustic emission (TEOAE) if the neonate passed tympanometry. Each ear was tested separately. If a neonate failed TEOAE testing in either ear, then automated auditory brainstem response (ABR) testing was performed. TEOAE and ABR testing were repeated at follow-up visits at the ages of 3 and 6 months. Children with abnormal hearing screening tests were referred locally for care.

### Statistical analysis

Data were collected using REDCap [15] and analyzed using STATA 16 (College Station, USA). Continuous variables were described using means and standard deviations (SD), and categorical variables were described using frequencies and proportions. We compared maternal and neonatal characteristics between cCMV-positive and cCMV-negative newborns using bivariate analyses. We also performed an exploratory logistic regression analysis to test for associations between maternal and neonatal characteristics and cCMV positivity.

### Ethical considerations

All participants provided written informed consent before participating in any study activity and the study was conducted in accordance with the ethical standards laid down in the 1964 Declaration of Helsinki and its later amendments. It was approved by the Mbarara University Research Ethics Committee (10/06–19), the Uganda National Council for Science and Technology (HS/2697) and the Partners (Mass General Brigham) IRB (2019P003248.

## Results

A total of 793 women and their newborns enrolled in the parent study were included in this sub-study. The mean maternal age at delivery was 28 (standard deviation [SD] 6) years, and the mean parity was 3 (SD 2). Overall, 726/793 (92%) of participants were married, the median monthly income was 42 (interquartile range [IQR] 22–94) USD, and 41% delivered by caesarean section. The mean birth weight was 3.0 (SD, 0.5) kilograms, and the mean gestational age at birth was 37 (SD, 2) weeks (Table 1).

**Table 1:** Overall maternal and infant characteristics of study participants (*N* = 793)

*Variable*	*Overall*,* n(%)*
**Maternal characteristics**	
Age in years, mean [SD]	28 [6.0]
Formally employed	534 [67.0]
Monthly income in USD, median [IQR]	42 [22.9]
Married	726 [92.0]
Attended at least 4 ANC visits	626 [79.0]
Cesarean delivery	321 [41.0]
Parity, mean [SD]	3 [2.0]
**Asset index percentile**	
Richest	197 [25.0]
Richer	194 [25.0]
Poorer	199 [25.0]
Poorest	198 [25.0]
**Neonatal characteristics**	
Birth weight (kg), mean [SD]	3 [0.5]
Weight < 2.5 kg	47 [6.0]
Gestational age (weeks), mean [SD]	37 [2.0]
Preterm delivery	82 [10.5]
Apgar < = 7 at 5 min	18 [2.0]
CMV positivity	23 [3.0]

SD= standard deviation, IQR= interquartile range, ANC= antenatal care

Overall, 677 out of 793 (84.7%) neonatal saliva samples were tested for cCMV, and 23 out of 677 (3.4%, CI 0.02–0.05) were positive. Cycle threshold results from 30 samples tested at a local reference laboratory were very similar to those of the primary lab, with no samples reclassified from cCMV negative to positive or vice versa in reference lab testing. In bivariate analyses, the 1-minute Apgar score of < 7, presence of neonatal jaundice, and failure of at least one hearing screen were associated with cCMV infection (Table 2).

Only 141 newborns and infants underwent at least one hearing screening. Specifically, 66 underwent hearing screening in the neonatal period, 38 underwent screening in the neonatal period and at 3 months, and 66 were screened in the neonatal period at 3 and 6 months. Of the infants who underwent at least one hearing test, 55/141 (39%) failed. None of the neonates or infants at 3 months or 6 months with cCMV failed a hearing test (Table 2).

In exploratory adjusted analyses, maternal HIV status (adjusted odds ratio (aOR) = 0.7, 95% confidence interval (CI) [0.3–1.7], *P* = 0.08), and gestational age (aOR = 0.9, 95% CI [0.9-1.0], *P* = 0.39), were not associated with cCMV positivity (Table 3).

**Table 2:** Maternal and newborn characteristics and their association with CMV saliva test results (*n* = 677)

Baseline characteristic	cCMV positive (*n* = 23)	cCMV negative (*n* = 654)	*P*-value
**Maternal characteristics**			
Maternal age categories in years			0.79
18–19	2 [8.7]	39 [5.9]	
20–34	18 [78.4]	508 [77.7]	
>34	3[13.0]	107 [16.4]	
Parity, mean (SD)			0.23
Primigravida	8 [34.8]	162 [24.9]	
Multipara (1–4)	10 [43.5]	390 [59.8]	
Grand multipara (> 5)	5 [21.7]	100 [15.3]	
HIV seropositive	12 [52.1]	332 [50.8]	0.53
Caesarean delivery	8 [34.8]	264 [41.2]	0.67
**Asset index quartiles**			
Poorest	8 [34.8]	162 [24.9]	0.32
Poor	8 [34.8]	160 [24.6]	
Richer	4 [17.4]	162 [24.9]	
Richest	3 [13.0]	166 [25.5]	
**Newborn characteristics**			
Birth weight < 2.5 kg	2 [8.7]	34 [5.2]	0.35
Neonate sex			0.09
Female	7 [30.4]	324 [49.6]	
Male	16 [69.6]	329 [50.4]	
**Gestational age corrected weight**			0.79
<10th centile	11 [52.4]	231 [35.7]	
10-25th centile	2 [9.5]	104 [16.1]	
26th -50th centile	3 [14.3]	117 [18.1]	
51st -75th centile	0[0.0]	90[13.9]	
76th -90th centile	2[9.5]	[6.9]45	
>90th centile	3 [14.3]	60 [9.3]	
Prematurity (gestational age < 37 weeks at birth)	2 [9.5]	50 [7.7]	0.67
Apgar < 7 at 1 min	3 [13.0]	26 [3.9]	0.07
Apgar < 7 at 5 min	0 [0.0]	3 [0.5]	1.0
**Neonatal clinical findings**			
Jaundice	1 [4.4]]	0 [0.0]	0.03*
Lethargy	1 [4.4]	6 [0.9]	0.2
Fever	0 [0.0]	17 [2.6]	1.00
Convulsions	0 [0]	1 [0.2]	0.96
Difficulty breathing	1 [4.4]	18 [2.8]	0.49
Poor feeding	0 [0]	14 [2.1]	1.0
Birth admission	2 [8.7]	50 [7.7]	0.69
**Hearing screening outcome** ^**b**^			
Failed hearing screen at birth	0 [0.0]	33 [82.5]	0.19
Failed hearing screen at 3 months	0 [0.0]	9 [39.1]	1.00
Failed hearing screen at 6 months	0 [0.0]	18 [37.5]	0.54
Failed any hearing screen	0 [0.0]	55 [54.5]	0.05*
Failed more than one hearing screen	0 [0.0]	25 [35.2]	0.29

b *n* = 141

* statistically significant P-value ≤ 0.05

**Table 3:** Exploratory logistic regression analysis of factors associated with cCMV, *n* = 677

Factors associated with neonatal cCMV	Adjusted odds ratio (95% confidence interval)	*P*-value
Gestational age at birth	0.9 (0.9-1.0)	0.08
Maternal HIV serostatus	0.7 (0.3–1.7)	0.39

## Discussion

We determined the prevalence of cCMV infection among HIV-exposed and -unexposed neonates, and the relationship between maternal HIV infection, infant characteristics, and cCMV infection in Uganda. Our findings revealed an overall cCMV prevalence of 3.4% (CI 0.02–0.05). Our findings align with the range of 0.6–6.1% reported in a meta-analysis of studies conducted in LMICs [16, 17]. Our study is an important addition to the literature because the meta-analysis did not differentiate cCMV infection rates based on HIV exposure status.

Although the overall prevalence of cCMV was higher than that reported in high-income countries, in contrast to previous studies that showed a higher risk of cCMV infection among HIV-exposed neonates, we found similar cCMV prevalence between HIV-exposed (3.4.%) and HIV-unexposed (3.3%) neonates. A Zambian-based study reported slightly higher cCMV prevalence among neonates admitted to a referral facility compared to our findings (3.4% vs. 4.5%). However, in this Zambian cohort, the prevalence of cCMV among HIV-exposed neonates was significantly higher (11.5%) than what we observed [6]. This discrepancy in cCMV prevalence between our cohort and the Zambia study may be attributed to the fact that the Zambian infants were recruited from those admitted and receiving inpatient care at a referral facility and likely represented a more severely ill cohort and also the fact that this cohort was recruited in 2014 and may have had less ART coverage since this was close to the roll out of the Option B+ treatment option for women. In contrast, the neonates in our study were included when their mothers were admitted for routine delivery, and few required admission to the pediatric ward or neonatal unit (55, 7.7%), and almost all the mothers were on ART.

In our bivariate analyses, we identified a significant association between cCMV infection and both the 1-minute Apgar score and neonatal jaundice, although the number of participants experiencing these outcomes was low, with only 1 case of documented neonatal jaundice and 3 participants with low 1-minute Apgar scores in the CMV-positive group; this likely represents an under-diagnosis since not all newborns were rigorously examined and chart documentation is often lacking. Only 5–10% of newborns with cCMV will display symptoms at birth, which can include jaundice, lethargy, and difficulty breathing, skin rash, and hepatosplenomegaly [7, 18]. In our cohort, 1/23 (4.3%) neonates with cCMV exhibited jaundice, and no cCMV-negative neonates were jaundiced (0/654, 0%, *P* = 0.03). The association between cCMV and low 1-minute Apgar score may reflect lethargy and difficulty breathing, sometimes seen with cCMV. 3/23 (13%) neonates with cCMV had a 1-minute Apgar score of < 7, while 26/654 (3.9%, *P* = 0.07) cCMV-negative neonates had an Apgar score of less than 7.

We did not find a significant relationship between cCMV infection and failed hearing screening. Although cCMV is recognized as the most common congenital cause of hearing loss [19], cCMV-related hearing impairments may manifest at birth or later in life and may progress during early childhood [20]. Some neonates and infants in this cohort who had normal hearing testing could potentially develop hearing loss beyond age 9 months, when we stopped our screening assessments. Additionally, accuracy hearing screening results is challenging for neonates and infants, resulting in low test sensitivity in this age group and potential for under-detecting of hearing loss. Notably, only about 20% of infants who were screened for cCMV underwent a hearing screen, and not all infants who were screened at enrollment had a 3- and/or 6-month follow-up screen.

### Hearing screen implementation challenges

During the study period, the hearing screen machine required factory calibration and resetting twice, resulting in missed screens. Furthermore, we faced additional challenges with the hearing screen due to a noisier-than-ideal environment for hearing screening. These challenges highlight some of the implementation challenges that may limit the application of hearing screening.

Our study has multiple strengths, including the large sample size, with a nearly equal number of HIV-exposed and -unexposed children, and longitudinal follow-up of enrolled mother-child dyads to link cCMV testing with clinical outcomes. We also collected rich demographic and clinical data and performed hearing screening on a large number of children. However, the study also has its limitations. First, neonates were not examined by a clinician specifically to identify signs and symptoms of cCMV (e.g., chorioretinitis, hepatosplenomegaly), which may have resulted in missed symptoms of cCMV. Additionally, hearing screening was not initiated at the start of the study, resulting in incomplete hearing screening data for all participants, also during the study period, the hearing screen machine required factory calibration and resetting twice, resulting in missed screens. Lastly, as we did not conduct testing of urine to confirm the diagnosis of cCMV, there may be some false positive saliva results. We tried to minimize false positive saliva results by using a strict cycle threshold cutoff of PCR results and testing 5% of samples at a local reference lab, with excellent agreement of results between laboratories.

In conclusion, our findings revealed a notably high prevalence of cCMV among both HIV-exposed and -unexposed neonates born in southwestern Uganda, with no difference based on HIV exposure status. Few symptoms were noted in the charts of neonates with cCMV, and hearing screening outcomes were similar based on cCMV status. These results may be generalizable to similar settings.

Future studies should include a comprehensive clinical and audiological assessment for both HIV-exposed and -unexposed neonates at birth and longitudinal follow-up to define the prevalence of CMV-associated hearing loss and other sequelae.

## Supplementary Information

Below is the link to the electronic supplementary material.

Supplementary Material 1

## Data Availability

The data supporting the findings of this study are available upon request from the corresponding author. The data are not publicly available due to concerns regarding the privacy of research participants **.**.

## Abbreviations

CMV: Cytomegalovirus
cCMV: Congenital cytomegalovirus
sSA: Sub-Saharan Africa
PCR: Polymerase Chain Reaction
SD: Standard Deviation
IQR: Interquartile Range
PACO: Placenta, Antibodies and Child Outcomes
MRRH: Mbarara Regional Referral Hospital
KCRC: Kabwohe Clinical Research Center
TEOAE: Transitory evoked otoacoustic emission
ABR: Auditory brainstem response
ANC: Antenatal Care
