# Supplementary material for: Congenital cytomegalovirus and associations with maternal HIV serostatus and child health outcomes in Uganda
Source: BMC Infect Dis. 2026 May 28;26:1400. doi: 10.1186/s12879-026-13655-2 (PMC13411582; doi:10.1186/s12879-026-13655-2)
Supplement: Supplementary file 1 — Supplementary Material 1 [file 12879_2026_13655_MOESM1_ESM.pdf]

Interview

Study ID

____

DEMOGRAPHICS

Say: "I would like to ask you some questions about where you live and information about your family. Please answer as honestly and completely as you can."

Ask the participant for this information prior to hospital discharge.

Date this form was completed

____

What is your age?

____
(Age in years)

Do you know your date of birth (the year, month OR day)?

◯ Yes
◯ No
(Select "Yes" if she knows ANY of: day, month OR year)

What is your date of birth?

____
(Write '01' for the day and/or month, if unknown, and 2900 for the year, if unknown)

What is the highest level of school you completed?

◯ P1-P6
◯ P7
◯ S1-S3
◯ S4
◯ S5
◯ S6
◯ Tertiary/vocational
◯ University
◯ Postgraduate
◯ No school
◯ Unknown
◯ Preferred not to answer

Do you personally own a phone?

◯ Yes
◯ No

What type of phone do you own?

☐ Mobile phone
☐ Smart phone

Do others in your household own any phones?

◯ Yes
◯ No

What type of phone do others in your household own?

☐ Mobile phone
☐ Smart phone

What is the main ACTIVITY or JOB you do to provide for your family/household - <br>- either jobs you are paid for or for which you are given other goods or services?	◯ Housewife<br>◯ Student<br>◯ Teacher<br>◯ Housekeeper (keeps house for people other than her own family)<br>◯ Local brew seller / bar or restaurant attendant<br>◯ Selling goods (direct interaction with customers)<br>◯ Business person (other than selling goods)<br>◯ Government / clerical / secretarial<br>◯ Mechanic<br>◯ Health Care worker<br>◯ Sex worker<br>◯ Trader<br>◯ Technician / artisan (plumber, welder, electrician)<br>◯ Military / police / security<br>◯ Trucker/driver/conductor<br>◯ Farmer (subsistence - working on her own land to provide for herself and her family)<br>◯ Farmer (commercial, agro, animal husbandry, etc)<br>◯ Accountant / banker<br>◯ Unemployed / None<br>◯ Other<br>(*chose only one main job)
"Other" main job or employment activity	__________________________________<br>(Write in other main job/employment)
Can the participant estimate her household income?	◯ Yes<br>◯ No
Considering ALL sources of income from YOUR HOUSEHOLD, what is your average household income PER MONTH over the past 3 months?	__________________________________<br>(*Write average monthly income in Ugandan Shillings WITHOUT commas or punctuation, for example 150000)

SAVE YOUR WORK!

How much does it cost for you to travel from your house to this hospital and then back - in other words, round-trip? Include the cost of any overnight stays.	__________________________________<br>(Enter amount in Ugandan Shillings (with NO commas, punctuation or spaces, for example 20000))
What is your primary means of transportation to hospital?	◯ Walking<br>◯ Bicycle<br>◯ Public Bus<br>◯ Motorbike<br>◯ Public taxi<br>◯ Special hire taxi<br>◯ Car owned by you or a household member<br>(If transportation is evenly split between 2 or more means, then choose most expensive means)
For this study, we define being married as being legally married or in a relationship as if married.<br><br>We call the person to whom you are married your spouse or partner.<br><br>Are you currently married, widowed, divorced or separated, or have you never been married?	◯ Married<br>◯ Widowed<br>◯ Divorced or separated<br>◯ Never married<br>(Choose the one best answer)

What is the highest level of school your spouse or partner completed?	◯ P1-P6<br>◯ P7<br>◯ S1-S3<br>◯ S4<br>◯ S5<br>◯ S6<br>◯ Tertiary/vocational<br>◯ University<br>◯ Postgraduate<br>◯ No school<br>◯ Unknown/unsure<br>(*This question refers to her husband or partner's level of education completed)
Where do you live? Choose the correct DISTRICT below:	◯ Mbarara<br>◯ Bushenyi<br>◯ Isingiro<br>◯ Sheema<br>◯ Rubirizi<br>◯ Ibanda<br>◯ Ntungamo<br>◯ Lyantonde<br>◯ Kiruhura<br>◯ Kabale<br>◯ Other
Other DISTRICT of residence	______________________________<br>(Write in other district of residence)
Where do you live? Choose the correct COUNTY within MBARARA DISTRICT below:	◯ Mbarara Municipality<br>◯ Rwampara<br>◯ Kashari<br>◯ Other
'Other' COUNTY within MBARARA DISTRICT	______________________________<br>(Write in 'other' county)
Where do you live? Choose the correct SUBCOUNTY within MBARARA DISTRICT below:	◯ Bubaare<br>◯ Bugamba<br>◯ Bukiro<br>◯ Kagongi<br>◯ Kakiika<br>◯ Kakoba<br>◯ Kamukuzi<br>◯ Kashare<br>◯ Mwizi<br>◯ Ndaija<br>◯ Nyakayojo<br>◯ Nyamitanga<br>◯ Rubaya<br>◯ Rubindi<br>◯ Rugando<br>◯ Rwanyamahembe<br>◯ Other
'Other' SUBCOUNTY within MBARARA DISTRICT	______________________________<br>(Write in 'other' sub county)

Where do you live? Choose the correct COUNTY within BUSHENYI DISTRICT below:	◯ Bushenyi-Ishaka Municipality<br>◯ Igara<br>◯ Other
'Other' COUNTY within BUSHENYI DISTRICT	__________________________________<br>(Write in 'other' county)
Where do you live? Choose the correct SUBCOUNTY within BUSHENYI DISTRICT below:	◯ Bumbaire<br>◯ Bushenyi-ishaka Tc<br>◯ Kakanju<br>◯ Kyabugimbi<br>◯ Kyamuhunga<br>◯ Kyeizoba<br>◯ Nyabubare<br>◯ Other
'Other' SUBCOUNTY within BUSHENYI DISTRICT	__________________________________<br>(Write in 'other' sub county)
Where do you live? Choose the correct COUNTY within ISINGIRO DISTRICT below:	◯ Isingiro North<br>◯ Isingiro South<br>◯ Bukanga<br>◯ Other
'Other' COUNTY within ISINGIRO DISTRICT	__________________________________<br>(Write in 'other' county)
Where do you live? Choose the correct SUBCOUNTY within ISINGIRO DISTRICT below:	◯ Birere<br>◯ Endinzi<br>◯ Kabingo<br>◯ Kabuyanda<br>◯ Kashumba<br>◯ Kikagate<br>◯ Masha<br>◯ Ngarama<br>◯ Nyakitunda<br>◯ Rugaaga<br>◯ Other
'Other' SUBCOUNTY within ISINGIRO DISTRICT	__________________________________<br>(Write in 'other' sub county)
Where do you live? Choose the correct COUNTY within SHEEMA DISTRICT below:	◯ Sheema North<br>◯ Sheema South<br>◯ Other
'Other' COUNTY within SHEEMA DISTRICT	__________________________________<br>(Write in 'other' county)

Where do you live? Choose the correct SUBCOUNTY within SHEEMA DISTRICT below:	◯ Kabwohe-Itendero Town Council<br>◯ Kigarama<br>◯ Kyangyenyi<br>◯ Shuuku<br>◯ Bugongi<br>◯ Kagango<br>◯ Kitagata<br>◯ Other
'Other' SUBCOUNTY within SHEEMA DISTRICT	____________________<br>(Write in 'other' sub county)
Where do you live? Choose the correct COUNTY within RUBIRIZI DISTRICT below:	◯ Bunyaruguru<br>◯ Katerera<br>◯ Other
'Other' COUNTY within RUBIRIZI DISTRICT	____________________<br>(Write in 'other' county)
Where do you live? Choose the correct SUBCOUNTY within RUBIRIZI DISTRICT below:	◯ Katunguru<br>◯ Kichwamba<br>◯ Rutoto<br>◯ Magambo<br>◯ Katerera<br>◯ Ryeru<br>◯ Kirugu<br>◯ Kyabakara<br>◯ Katanda<br>◯ Rubirizi Town Council<br>◯ Other
'Other' SUBCOUNTY within RUBIRIZI DISTRICT	____________________<br>(Write in 'other' sub county)
Where do you live? Choose the correct COUNTY within IBANDA DISTRICT below:	◯ Ibanda North<br>◯ Ibanda South<br>◯ Other
'Other' COUNTY within IBANDA DISTRICT	____________________<br>(Write in 'other' county)
Where do you live? Choose the correct SUBCOUNTY within IBANDA DISTRICT below:	◯ Bisheshe<br>◯ Ibanda Tc<br>◯ Ishongororo<br>◯ Kicuzi<br>◯ Kikyenkye<br>◯ Nyabuhikye<br>◯ Nyamarebe<br>◯ Rukiri<br>◯ Other
'Other' SUBCOUNTY within IBANDA DISTRICT	____________________<br>(Write in 'other' sub county)

Where do you live? Choose the correct COUNTY within NTUNGAMO DISTRICT below:	◯ Ruhama<br>◯ Ntungamo Municipality<br>◯ Kazaara<br>◯ Other
'Other' COUNTY within NTUNGAMO DISTRICT	__________________<br>(Write in 'other' county)
Where do you live? Choose the correct SUBCOUNTY within NTUNGAMO DISTRICT below:	◯ Bwongyera<br>◯ Ihunga<br>◯ Itojo<br>◯ Kayonza<br>◯ Kibatsi<br>◯ Ngoma<br>◯ Ntungamo<br>◯ Ntungamo Tc<br>◯ Nyabihoko<br>◯ Nyakyera<br>◯ Rubaare<br>◯ Rugarama<br>◯ Ruhaama<br>◯ Rukoni<br>◯ Rweikiniro<br>◯ Other
'Other' SUBCOUNTY within NTUNGAMO DISTRICT	__________________<br>(Write in 'other' sub county)
Where do you live? Choose the correct COUNTY within LYANTONDE DISTRICT:	◯ Kabula<br>◯ Other
'Other' COUNTY within LYANTONDE DISTRICT	__________________<br>(Write in 'other' county)
Where do you live? Choose the correct SUBCOUNTY within LYANTONDE DISTRICT below:	◯ Kaliiro<br>◯ Kasagama<br>◯ Kinuuka<br>◯ Lyantonde<br>◯ Lyantonde Tc<br>◯ Mpumudde<br>◯ Other
'Other' SUBCOUNTY within LYANTONDE DISTRICT	__________________<br>(Write in 'other' sub county)
Where do you live? Choose the correct COUNTY within KIRUHURA DISTRICT:	◯ Kazo<br>◯ Nyabushozi<br>◯ Other
'Other' COUNTY within KIRUHURA DISTRICT	__________________<br>(Write in 'other' county)

Where do you live? Choose the correct SUBCOUNTY within KIRUHURA DISTRICT:

- ◯ Buremba
- ◯ Burunga
- ◯ Kanoni
- ◯ Kanyaryeru
- ◯ Kashongi
- ◯ Kazo
- ◯ Kenshunga
- ◯ Kikatsi
- ◯ Kinoni
- ◯ Nyakashashara
- ◯ Rwemikoma
- ◯ Sanga
- ◯ Other

'Other' SUBCOUNTY within KIRUHURA DISTRICT

____
(Write in 'other' sub county)

Where do you live? Choose the correct COUNTY within KABALE DISTRICT:

- ◯ Kabale Municipality
- ◯ Rubanda
- ◯ Rukiga
- ◯ Ndorwa
- ◯ Other

'Other' COUNTY within KABALE DISTRICT

____
(Write in 'other' county)

Where do you live? Choose the correct SUBCOUNTY within KABALE DISTRICT below:

- ◯ Bubare
- ◯ Bufundi
- ◯ Buhara
- ◯ Bukinda
- ◯ Hamurwa
- ◯ Ikumba
- ◯ Kabale Central
- ◯ Kabale Northern
- ◯ Kabale Southern
- ◯ Kaharo
- ◯ Kamuganguzi
- ◯ Kamwezi
- ◯ Kashambya
- ◯ Kitumba
- ◯ Kyanamira
- ◯ Maziba
- ◯ Muko
- ◯ Rubaya
- ◯ Rwamucucu
- ◯ Other

'Other' SUBCOUNTY within KABALE DISTRICT

____
(Write in 'other' sub county)

Where do you live? Write in the correct DIVISION.

____
(Write the name of the divison where the participant lives. Write 'unknown' or 'unk' if she does not know her division.)

Where do you live? Write in the correct PARISH.

____
(Write the name of the parish where the participant lives. Write 'unknown' or 'unk' if she does not know her parish.)

Where do you live? Write in the correct VILLAGE.

____
(Write the name of the village where the participant lives. Write 'unknown' or 'unk' if she does not know her village.)

Where do you live? Write in the correct ZONE.

____
(Write the name of the zone where the participant lives. Write 'unknown' or 'unk' if she does not know her zone.)

Where do you live? Write in the correct WARD.

____
(Write the name of the ward where the participant lives. Write 'unknown' or 'unk' if she does not know her ward.)

Where do you live? Write in the correct CELL.

____
(Write the name of the cell where the participant lives. Write 'unknown' of 'unk' if she does not know her cell.)

Who is the LC1 Chairman where you live and what is his/her phone number?

____
(Write in the NAME of the LC1 CHAIRPERSON and his/her phone number. Write 'unknown' or 'unk' if she does not know the name of the LC1 chair.)

What are the specific directions I would follow in order to reach the place where you live?

____

Now I will ask you some questions about your household. For this study, consider your household to include people with whom you usually live and share meals. Please include children who are in boarding school. If you live in your employer's household, please refer to your own family's household.

Do you or a member of your household own a house?

◯ Yes
◯ No

How many separate rooms are in your house, including all living areas, bathrooms, and any other rooms?

____
(Write in the number of separate rooms, for example '4'.)

How many adults (>18 years) live in your house, including yourself?

____
(Include the participant in the total.)

How many children (< 18 years) live in your house?	______________________ (Write '0' if there are no children living in the house)
Does the participant know whether her household pays monthly rent?	◯ Yes<br>◯ No
What amount, if any, does your household pay for monthly rent?	______________________ (Write in her best estimate in Ugandan Shillings, using NO commas or spaces. For example, write '100000')
What kind of toilet facility does your household use?	◯ None<br>◯ Pan/bucket<br>◯ Uncovered pit latrine<br>◯ Covered pit latrine<br>◯ VIP pit latrine<br>◯ Flush toilet<br>◯ Other<br>(Choose only the most commonly used type of toilet.)
'Other' type of toilet facility	______________________ (Write in 'other' type of toilet facility)
Is this toilet facility shared with other households?	◯ Yes<br>◯ No<br>◯ Unknown / Unsure
What is the roof of your house made of?	◯ Metal: tin<br>◯ Metal: iron<br>◯ Metal: other type or unknown metal type<br>◯ Grass/straw/papyrus/thatched<br>◯ Tiles<br>◯ Other
'Other' type of roof	______________________ (Write in 'other' type of roof)
What are the walls of your house made of?	◯ Mud/dirt wattle<br>◯ Mud/clay bricks<br>◯ Cement<br>◯ Other
'Other' type of walls	______________________ (Write in 'other' type of walls)
What is the floor of your house made of?	◯ Mud/dirt dung<br>◯ Mud/Dirt with covering<br>◯ Cement<br>◯ Vinyl<br>◯ Tiles<br>◯ Other

'Other' type of floor

____
(Write in 'other' type of floor)

Where do you get water?

- ◯ Piped into dwelling
- ◯ Communal tap
- ◯ Open well
- ◯ Protected well
- ◯ Protected stream - a constant flow of water out of an open pipe, in which the source is underground or protected
- ◯ Unprotected spring - a constant flow of water from an open natural stream without fencing to protect it from animals or children
- ◯ Stream - water flowing without a point source
- ◯ Public borehole
- ◯ Other

(Mark the PRIMARY water source)

'Other' source of water

____
(Write in 'other' source of water)

SAVE your work!

Do members of your household own any of the following items?

- ☐ Electric iron
- ☐ Stove with gas or electric burners
- ☐ Refrigerator or freezer
- ☐ Telephone
- ☐ Motorbike
- ☐ Clock
- ☐ Bed
- ☐ Sofa
- ☐ Bicycle
- ☐ Television
- ☐ Lantern
- ☐ Cupboard
- ☐ Shoes other than slippers
- ☐ Car
- ☐ Radio
- ☐ Mattress
- ☐ None of these items
- ☐ Refused to answer

(Mark all that apply, including ONLY those in working condition)

Do the members of the household own any land?

- ◯ Yes
- ◯ No
- ◯ Unknown / Unsure

Does your house have electricity?

- ◯ Yes
- ◯ No
- ◯ Unknown / Unsure

Did you use a mosquito net last night?

- ◯ Yes
- ◯ No

How many times did you use a mosquito net in the last 3 nights?

- ◯ 0
- ◯ 1
- ◯ 2
- ◯ 3

How many times did you use a mosquito net in the last 7 nights?

- ◯ 0
- ◯ 1
- ◯ 2
- ◯ 3
- ◯ 4
- ◯ 5
- ◯ 6
- ◯ 7

During the last one month, how often did you sleep under a mosquito net?

- ◯ None of the time
- ◯ A little of the time
- ◯ Some of the time
- ◯ A good bit of the time
- ◯ Most of the time
- ◯ All of the time

During THIS PREGNANCY, how often did you sleep under a mosquito net?

- ◯ None of the time
- ◯ A little of the time
- ◯ Some of the time
- ◯ A good bit of the time
- ◯ Most of the time
- ◯ All of the time

Is your mosquito net treated with insecticide?

- ◯ Yes
- ◯ No
- ◯ Unknown / Unsure
- ◯ I do not have a mosquito net

During this pregnancy, have you used any of the following?

--> MARK ALL THAT APPLY

	Used this pregnancy	Used in the last 30 days	Injected in your veins (EVER)	Injected in your veins (in the last 30 days)	Did not use at all this pregnancy
Alcohol	☐	☐	☐	☐	☐
Smoking tobacco or cigarettes	☐	☐	☐	☐	☐
MarjuANA	☐	☐	☐	☐	☐
Chewing tobacco	☐	☐	☐	☐	☐
Petrol for sniffing	☐	☐	☐	☐	☐
Paint thinner for sniffing	☐	☐	☐	☐	☐
Crack	☐	☐	☐	☐	☐
Cocaine	☐	☐	☐	☐	☐
Heroin	☐	☐	☐	☐	☐
Speed or methamphetamine	☐	☐	☐	☐	☐
MariJUNI	☐	☐	☐	☐	☐

SAVE your work!

During this pregnancy, how often did you have a drink containing alcohol?	◯ Never<br>◯ Monthly or less<br>◯ 2-4 times a month<br>◯ 2-3 times a week<br>◯ 4 or more times a week<br>◯ Don't know<br>◯ Decline to answer
During this pregnancy, how many drinks of any kind containing alcohol did you have on a typical day when you were drinking?	◯ 1 or 2<br>◯ 3 or 4<br>◯ 5 or 6<br>◯ 7, 8 or 9<br>◯ 10 or more<br>◯ Cannot estimate because of use of non-standardized, non-bottled, home-brewed beverages<br>◯ Don't know<br>◯ Decline to answer
During this pregnancy, how often did you have six (6) or more alcohol-containing drinks on one occasion?	◯ Never<br>◯ Less than monthly<br>◯ Monthly<br>◯ Weekly<br>◯ Daily or almost daily<br>◯ Don't know<br>◯ Decline to answer
How often during this pregnancy have you found that you were not able to stop drinking alcohol-containing drinks once you had started?	◯ Never<br>◯ Less than monthly<br>◯ Monthly<br>◯ Weekly<br>◯ Daily or almost daily<br>◯ Don't know<br>◯ Decline to answer
How often during this pregnancy have you failed to do what was normally expected of you because of drinking alcohol?	◯ Never<br>◯ Less than monthly<br>◯ Monthly<br>◯ Weekly<br>◯ Daily or almost daily<br>◯ Don't know<br>◯ Decline to answer
How often during this pregnancy have you needed a first alcohol-containing drink in the morning to get yourself going after a heavy drinking session?	◯ Never<br>◯ Less than monthly<br>◯ Monthly<br>◯ Weekly<br>◯ Daily or almost daily<br>◯ Don't know<br>◯ Decline to answer
How often during this pregnancy have you had a feeling of guilt or remorse after drinking alcohol?	◯ Never<br>◯ Less than monthly<br>◯ Monthly<br>◯ Weekly<br>◯ Daily or almost daily<br>◯ Don't know<br>◯ Decline to answer

How often during this pregnancy have you been unable to remember what happened the night before because you had been drinking alcohol?	◯ Never<br>◯ Less than monthly<br>◯ Monthly<br>◯ Weekly<br>◯ Daily or almost daily<br>◯ Don't know<br>◯ Decline to answer
Have you or someone else ever been injured as a result of your drinking alcohol?	◯ No<br>◯ Yes<br>◯ Don't know<br>◯ Decline to answer
When did this (injury) occur?	◯ In the past 3 months<br>◯ In the past 3-12 months<br>◯ Over a year ago<br>◯ Don't know<br>◯ Decline to answer
Has a relative or friend or a doctor or other health worker ever in your life been concerned about your drinking or suggested you cut down?	◯ No<br>◯ Yes<br>◯ Don't know<br>◯ Decline to answer
When was this?	◯ In the past 3 months<br>◯ In the past 3-12 months<br>◯ Over a year ago<br>◯ Don't know<br>◯ Decline to answer
Who was concerned about your drinking or suggested you cut down?	☐ Spouse/partner(s)<br>☐ Child(ren)<br>☐ Female family member(s)<br>☐ Male family member(s)<br>☐ Female friend(s)<br>☐ Male friend(s)<br>☐ Someone at work or school<br>☐ A doctor or healthcare worker<br>☐ Other<br>☐ Don't know<br>☐ Decline to answer
Who was concerned?	_________________________<br>(Write in the relationship of the concerned person to the participant)

Has a doctor or other medical care provider ever told you that you that you have any of the following conditions, excluding HIV?

	During this pregnancy	During another pregnancy	When not pregnant	NEVER
High blood pressure or hypertension	☐	☐	☐	☐

Pre-eclampia or eclampsia	☐	☐	☐	☐
Urinary tract infection	☐	☐	☐	☐
Malaria	☐	☐	☐	☐
Syphilis	☐	☐	☐	☐
Sexually transmitted infection (other than syphilis)	☐	☐	☐	☐
Diabetes	☐	☐	☐	☐
Heart condition	☐	☐	☐	☐
Kidney disease	☐	☐	☐	☐
Lupus or another autoimmune disorder	☐	☐	☐	☐
Tuberculosis (TB)	☐	☐	☐	☐
Asthma	☐	☐	☐	☐
Anemia (low blood counts)	☐	☐	☐	☐
Skin or wound infection	☐	☐	☐	☐
Vaginal infection (including candidiasis, bacterial vaginosis but NOT syphilis, chlamydia, gonorrhea or herpes)	☐	☐	☐	☐
Other (1st other)	☐	☐	☐	☐
Other (2nd other)	☐	☐	☐	☐
Other (3rd other)	☐	☐	☐	☐

1st other medical condition

____
(specify other medical condition (not HIV))

2nd other medical condition

____
(specify other medical condition (not HIV))

3rd other medical condition

____
(specify other medical condition (not HIV))

For high blood pressure, what type of treatment did you receive?

☐ Traditional (herbs, etc.)
☐ Medical
☐ Other type
☐ None
(*Select all types of treatment the participant received for this condition.)

For pre-eclampsia/eclampsia, what type of treatment did you receive?

☐ Traditional (herbs, etc.)
☐ Medical
☐ Other type
☐ None
(*Select all types of treatment the participant received for this condition.)

For urinary tract infection, what type of treatment did you receive?	☐ Traditional (herbs, etc.)<br>☐ Medical<br>☐ Other type<br>☐ None<br>(*Select all types of treatment the participant received for this condition.)
For malaria, what type of treatment did you receive?	☐ Traditional (herbs, etc.)<br>☐ Medical<br>☐ Other type<br>☐ None<br>(*Select all types of treatment the participant received for this condition.)
For syphilis, what type of treatment did you receive?	☐ Traditional (herbs, etc.)<br>☐ Medical<br>☐ Other type<br>☐ None<br>(*Select all types of treatment the participant received for this condition.)
For the other sexually transmitted infection what type of treatment did you receive?	☐ Traditional (herbs, etc.)<br>☐ Medical<br>☐ Other type<br>☐ None<br>(*Select all types of treatment the participant received for this condition.)
For diabetes, what type of treatment did you receive?	☐ Traditional (herbs, etc.)<br>☐ Medical<br>☐ Other type<br>☐ None<br>(*Select all types of treatment the participant received for this condition.)
For the heart condition, what type of treatment did you receive?	☐ Traditional (herbs, etc.)<br>☐ Medical<br>☐ Other type<br>☐ None<br>(*Select all types of treatment the participant received for this condition.)
For kidney disease, what type of treatment did you receive?	☐ Traditional (herbs, etc.)<br>☐ Medical<br>☐ Other type<br>☐ None<br>(*Select all types of treatment the participant received for this condition.)
For lupus or other autoimmune disorder, what type of treatment did you receive?	☐ Traditional (herbs, etc.)<br>☐ Medical<br>☐ Other type<br>☐ None<br>(*Select all types of treatment the participant received for this condition.)

For tuberculosis (TB), what type of treatment did you receive?	☐ Traditional (herbs, etc.)<br>☐ Medical<br>☐ Other type<br>☐ None<br>(*Select all types of treatment the participant received for this condition.)
For asthma, what type of treatment did you receive?	☐ Traditional (herbs, etc.)<br>☐ Medical<br>☐ Other type<br>☐ None<br>(*Select all types of treatment the participant received for this condition.)
For anemia, what type of treatment did you receive?	☐ Traditional (herbs, etc.)<br>☐ Medical<br>☐ Other type<br>☐ None<br>(*Select all types of treatment the participant received for this condition.)
For skin or wound infection, what type of treatment did you receive?	☐ Traditional (herbs, etc.)<br>☐ Medical<br>☐ Other type<br>☐ None<br>(*Select all types of treatment the participant received for this condition.)
For the vaginal infection, what type of treatment did you receive?	☐ Traditional (herbs, etc.)<br>☐ Medical<br>☐ Other type<br>☐ None<br>(*Select all types of treatment the participant received for this condition.)
For 1st other medical condition, what type of treatment did you receive?	☐ Traditional (herbs, etc.)<br>☐ Medical<br>☐ Other type<br>☐ None<br>(*Select all types of treatment the participant received for this condition.)
For 2nd other medical condition, what type of treatment did you receive?	☐ Traditional (herbs, etc.)<br>☐ Medical<br>☐ Other type<br>☐ None<br>(*Select all types of treatment the participant received for this condition.)
For 3rd other medical condition, what type of treatment did you receive?	☐ Traditional (herbs, etc.)<br>☐ Medical<br>☐ Other type<br>☐ None<br>(*Select all types of treatment the participant received for this condition.)

*** For each pregnancy, write the correct information for when the pregnancy ENDED.**

How many pregnancies have you had in your life, whether or not they resulted in a live baby?

- ◯ 1
- ◯ 2
- ◯ 3
- ◯ 4
- ◯ 5
- ◯ 6
- ◯ 7
- ◯ 8
- ◯ 9
- ◯ 10
- ◯ 11
- ◯ 12
- ◯ 13
- ◯ 14
- ◯ 15
- ◯ 16
- ◯ 17
- ◯ 18
- ◯ 19
- ◯ 20

(Include the current/recent pregnancy in the total count.)

What year was the 1st pregnancy?

____

(Write '01' for the day and/or month, if unknown, and 2900 for the year, if unknown)

How far along were you when the 1st pregnancy ended?

- ◯ Before 12 weeks' gestation (early miscarriage)
- ◯ 12-28 weeks' gestation (late miscarriage)
- ◯ 28-32 weeks' gestation (very preterm)
- ◯ 32-36 weeks' gestation (preterm)
- ◯ 37-40 weeks' gestation (term)
- ◯ After 40 weeks' gestation (post-dates)
- ◯ Unknown/unsure

What type of delivery did you have for the 1st pregnancy?

- ◯ Vaginal (normal)
- ◯ Cesarean

Was the baby (or babies) from the 1st pregnancy born alive?

- ◯ Yes
- ◯ No
- ◯ Unknown/unsure
- ◯ One baby born alive, one or more born dead

Did the the baby (or babies) from the 1st pregnancy die within the first 30 days (1 month) after birth?

- ◯ Yes
- ◯ No
- ◯ Unknown/unsure
- ◯ One baby alive, one or more died

What year was the 2nd pregnancy?

____

(Write '01' for the day and/or month, if unknown, and 2900 for the year, if unknown)

How far along were you when the 2nd pregnancy ended?	◯ Before 12 weeks' gestation (early miscarriage)<br>◯ 12-28 weeks' gestation (late miscarriage)<br>◯ 28-32 weeks' gestation (very preterm)<br>◯ 32-36 weeks' gestation (preterm)<br>◯ 37-40 weeks' gestation (term)<br>◯ After 40 weeks' gestation (post-dates)<br>◯ Unknown/unsure
What type of delivery did you have for the 2nd pregnancy?	◯ Vaginal (normal)<br>◯ Cesarean
Was the baby (or babies) from the 2nd pregnancy born alive?	◯ Yes<br>◯ No<br>◯ Unknown/unsure<br>◯ One baby born alive, one or more born dead
Did the the baby (or babies) from the 2nd pregnancy die within the first 30 days (1 month) after birth?	◯ Yes<br>◯ No<br>◯ Unknown/unsure<br>◯ One baby alive, one or more died
What year was the 3rd pregnancy?	__________________________________<br>(Write '01' for the day and/or month, if unknown, and 2900 for the year, if unknown)
How far along were you when the 3rd pregnancy ended?	◯ Before 12 weeks' gestation (early miscarriage)<br>◯ 12-28 weeks' gestation (late miscarriage)<br>◯ 28-32 weeks' gestation (very preterm)<br>◯ 32-36 weeks' gestation (preterm)<br>◯ 37-40 weeks' gestation (term)<br>◯ After 40 weeks' gestation (post-dates)<br>◯ Unknown/unsure
What type of delivery did you have for the 3rd pregnancy?	◯ Vaginal (normal)<br>◯ Cesarean
Was the baby (or babies) from the 3rd pregnancy born alive?	◯ Yes<br>◯ No<br>◯ Unknown/unsure<br>◯ One baby born alive, one or more born dead
Did the the baby (or babies) from the 3rd pregnancy die within the first 30 days (1 month) after birth?	◯ Yes<br>◯ No<br>◯ Unknown/unsure<br>◯ One baby alive, one or more died
What year was the 4th pregnancy?	__________________________________<br>(Write '01' for the day and/or month, if unknown, and 2900 for the year, if unknown)
How far along were you when the 4th pregnancy ended?	◯ Before 12 weeks' gestation (early miscarriage)<br>◯ 12-28 weeks' gestation (late miscarriage)<br>◯ 28-32 weeks' gestation (very preterm)<br>◯ 32-36 weeks' gestation (preterm)<br>◯ 37-40 weeks' gestation (term)<br>◯ After 40 weeks' gestation (post-dates)<br>◯ Unknown/unsure

What type of delivery did you have for the 4th pregnancy?	◯ Vaginal (normal)<br>◯ Cesarean
Was the baby (or babies) from the 4th pregnancy born alive?	◯ Yes<br>◯ No<br>◯ Unknown/unsure<br>◯ One baby born alive, one or more born dead
Did the the baby (or babies) from the 4th pregnancy die within the first 30 days (1 month) after birth?	◯ Yes<br>◯ No<br>◯ Unknown/unsure<br>◯ One baby alive, one or more died
What year was the 5th pregnancy?	________________________________<br>(Write '01' for the day and/or month, if unknown, and 2900 for the year, if unknown)
How far along were you when the 5th pregnancy ended?	◯ Before 12 weeks' gestation (early miscarriage)<br>◯ 12-28 weeks' gestation (late miscarriage)<br>◯ 28-32 weeks' gestation (very preterm)<br>◯ 32-36 weeks' gestation (preterm)<br>◯ 37-40 weeks' gestation (term)<br>◯ After 40 weeks' gestation (post-dates)<br>◯ Unknown/unsure
What type of delivery did you have for the 5th pregnancy?	◯ Vaginal (normal)<br>◯ Cesarean
Was the baby (or babies) from the 5th pregnancy born alive?	◯ Yes<br>◯ No<br>◯ Unknown/unsure<br>◯ One baby born alive, one or more born dead
Did the the baby (or babies) from the 5th pregnancy die within the first 30 days (1 month) after birth?	◯ Yes<br>◯ No<br>◯ Unknown/unsure<br>◯ One baby alive, one or more died
What year was the 6th pregnancy?	________________________________<br>(Write '01' for the day and/or month, if unknown, and 2900 for the year, if unknown)
How far along were you when the 6th pregnancy ended?	◯ Before 12 weeks' gestation (early miscarriage)<br>◯ 12-28 weeks' gestation (late miscarriage)<br>◯ 28-32 weeks' gestation (very preterm)<br>◯ 32-36 weeks' gestation (preterm)<br>◯ 37-40 weeks' gestation (term)<br>◯ After 40 weeks' gestation (post-dates)<br>◯ Unknown/unsure
What type of delivery did you have for the 6th pregnancy?	◯ Vaginal (normal)<br>◯ Cesarean
Was the baby (or babies) from the 6th pregnancy born alive?	◯ Yes<br>◯ No<br>◯ Unknown/unsure<br>◯ One baby born alive, one or more born dead

Did the the baby (or babies) from the 6th pregnancy die within the first 30 days (1 month) after birth?	◯ Yes<br>◯ No<br>◯ Unknown/unsure<br>◯ One baby alive, one or more died
What year was the 7th pregnancy?	__________________________________<br>(Write '01' for the day and/or month, if unknown, and 2900 for the year, if unknown)
How far along were you when the 7th pregnancy ended?	◯ Before 12 weeks' gestation (early miscarriage)<br>◯ 12-28 weeks' gestation (late miscarriage)<br>◯ 28-32 weeks' gestation (very preterm)<br>◯ 32-36 weeks' gestation (preterm)<br>◯ 37-40 weeks' gestation (term)<br>◯ After 40 weeks' gestation (post-dates)<br>◯ Unknown/unsure
What type of delivery did you have for the 7th pregnancy?	◯ Vaginal (normal)<br>◯ Cesarean
Was the baby (or babies) from the 7th pregnancy born alive?	◯ Yes<br>◯ No<br>◯ Unknown/unsure<br>◯ One baby born alive, one or more born dead
Did the the baby (or babies) from the 7th pregnancy die within the first 30 days (1 month) after birth?	◯ Yes<br>◯ No<br>◯ Unknown/unsure<br>◯ One baby alive, one or more died
What year was the 8th pregnancy?	__________________________________<br>(Write '01' for the day and/or month, if unknown, and 2900 for the year, if unknown)
How far along were you when the 8th pregnancy ended?	◯ Before 12 weeks' gestation (early miscarriage)<br>◯ 12-28 weeks' gestation (late miscarriage)<br>◯ 28-32 weeks' gestation (very preterm)<br>◯ 32-36 weeks' gestation (preterm)<br>◯ 37-40 weeks' gestation (term)<br>◯ After 40 weeks' gestation (post-dates)<br>◯ Unknown/unsure
What type of delivery did you have for the 8th pregnancy?	◯ Vaginal (normal)<br>◯ Cesarean
Was the baby (or babies) from the 8th pregnancy born alive?	◯ Yes<br>◯ No<br>◯ Unknown/unsure<br>◯ One baby born alive, one or more born dead
Did the the baby (or babies) from the 8th pregnancy die within the first 30 days (1 month) after birth?	◯ Yes<br>◯ No<br>◯ Unknown/unsure<br>◯ One baby alive, one or more died

What year was the 9th pregnancy?	__________________________________<br>(Write '01' for the day and/or month, if unknown, and 2900 for the year, if unknown)
How far along were you when the 9th pregnancy ended?	◯ Before 12 weeks' gestation (early miscarriage)<br>◯ 12-28 weeks' gestation (late miscarriage)<br>◯ 28-32 weeks' gestation (very preterm)<br>◯ 32-36 weeks' gestation (preterm)<br>◯ 37-40 weeks' gestation (term)<br>◯ After 40 weeks' gestation (post-dates)<br>◯ Unknown/unsure
What type of delivery did you have for the 9th pregnancy?	◯ Vaginal (normal)<br>◯ Cesarean
Was the baby (or babies) from the 9th pregnancy born alive?	◯ Yes<br>◯ No<br>◯ Unknown/unsure<br>◯ One baby born alive, one or more born dead
Did the the baby (or babies) from the 9th pregnancy die within the first 30 days (1 month) after birth?	◯ Yes<br>◯ No<br>◯ Unknown/unsure<br>◯ One baby alive, one or more died
What year was the 10th pregnancy?	__________________________________<br>(Write '01' for the day and/or month, if unknown, and 2900 for the year, if unknown)
How far along were you when the 10th pregnancy ended?	◯ Before 12 weeks' gestation (early miscarriage)<br>◯ 12-28 weeks' gestation (late miscarriage)<br>◯ 28-32 weeks' gestation (very preterm)<br>◯ 32-36 weeks' gestation (preterm)<br>◯ 37-40 weeks' gestation (term)<br>◯ After 40 weeks' gestation (post-dates)<br>◯ Unknown/unsure
What type of delivery did you have for the 10th pregnancy?	◯ Vaginal (normal)<br>◯ Cesarean
Was the baby (or babies) from the 10th pregnancy born alive?	◯ Yes<br>◯ No<br>◯ Unknown/unsure<br>◯ One baby born alive, one or more born dead
Did the the baby (or babies) from the 10th pregnancy die within the first 30 days (1 month) after birth?	◯ Yes<br>◯ No<br>◯ Unknown/unsure<br>◯ One baby alive, one or more died
What year was the 11th pregnancy?	__________________________________

How far along were you when the 11th pregnancy ended?	◯ Before 12 weeks' gestation (early miscarriage)<br>◯ 12-28 weeks' gestation (late miscarriage)<br>◯ 28-32 weeks' gestation (very preterm)<br>◯ 32-36 weeks' gestation (preterm)<br>◯ 37-40 weeks' gestation (term)<br>◯ After 40 weeks' gestation (post-dates)<br>◯ Unknown/unsure
What type of delivery did you have for the 11th pregnancy?	◯ Vaginal (normal)<br>◯ Cesarean
Was the baby (or babies) from the 11th pregnancy born alive?	◯ Yes<br>◯ No<br>◯ Unknown/unsure<br>◯ One baby born alive, one or more born dead
Did the the baby (or babies) from the 11th pregnancy die within the first 30 days (1 month) after birth?	◯ Yes<br>◯ No<br>◯ Unknown/unsure<br>◯ One baby alive, one or more died
What year was the 12th pregnancy?	______________________
How far along were you when the 12th pregnancy ended?	◯ Before 12 weeks' gestation (early miscarriage)<br>◯ 12-28 weeks' gestation (late miscarriage)<br>◯ 28-32 weeks' gestation (very preterm)<br>◯ 32-36 weeks' gestation (preterm)<br>◯ 37-40 weeks' gestation (term)<br>◯ After 40 weeks' gestation (post-dates)<br>◯ Unknown/unsure
What type of delivery did you have for the 12th pregnancy?	◯ Vaginal (normal)<br>◯ Cesarean
Was the baby (or babies) from the 12th pregnancy born alive?	◯ Yes<br>◯ No<br>◯ Unknown/unsure<br>◯ One baby born alive, one or more born dead
Did the the baby (or babies) from the 12th pregnancy die within the first 30 days (1 month) after birth?	◯ Yes<br>◯ No<br>◯ Unknown/unsure<br>◯ One baby alive, one or more died
What year was the 13th pregnancy?	______________________
How far along were you when the 13th pregnancy ended?	◯ Before 12 weeks' gestation (early miscarriage)<br>◯ 12-28 weeks' gestation (late miscarriage)<br>◯ 28-32 weeks' gestation (very preterm)<br>◯ 32-36 weeks' gestation (preterm)<br>◯ 37-40 weeks' gestation (term)<br>◯ After 40 weeks' gestation (post-dates)<br>◯ Unknown/unsure
What type of delivery did you have for the 13th pregnancy?	◯ Vaginal (normal)<br>◯ Cesarean

Was the baby (or babies) from the 13th pregnancy born alive?	◯ Yes<br>◯ No<br>◯ Unknown/unsure<br>◯ One baby born alive, one or more born dead
Did the the baby (or babies) from the 13th pregnancy die within the first 30 days (1 month) after birth?	◯ Yes<br>◯ No<br>◯ Unknown/unsure<br>◯ One baby alive, one or more died
What year was the 14th pregnancy?	______________________
How far along were you when the 14th pregnancy ended?	◯ Before 12 weeks' gestation (early miscarriage)<br>◯ 12-28 weeks' gestation (late miscarriage)<br>◯ 28-32 weeks' gestation (very preterm)<br>◯ 32-36 weeks' gestation (preterm)<br>◯ 37-40 weeks' gestation (term)<br>◯ After 40 weeks' gestation (post-dates)<br>◯ Unknown/unsure
What type of delivery did you have for the 14th pregnancy?	◯ Vaginal (normal)<br>◯ Cesarean
Was the baby (or babies) from the 14th pregnancy born alive?	◯ Yes<br>◯ No<br>◯ Unknown/unsure<br>◯ One baby born alive, one or more born dead
Did the the baby (or babies) from the 14th pregnancy die within the first 30 days (1 month) after birth?	◯ Yes<br>◯ No<br>◯ Unknown/unsure<br>◯ One baby alive, one or more died
What year was the 15th pregnancy?	______________________
How far along were you when the 15th pregnancy ended?	◯ Before 12 weeks' gestation (early miscarriage)<br>◯ 12-28 weeks' gestation (late miscarriage)<br>◯ 28-32 weeks' gestation (very preterm)<br>◯ 32-36 weeks' gestation (preterm)<br>◯ 37-40 weeks' gestation (term)<br>◯ After 40 weeks' gestation (post-dates)<br>◯ Unknown/unsure
What type of delivery did you have for the 15th pregnancy?	◯ Vaginal (normal)<br>◯ Cesarean
Was the baby (or babies) from the 15th pregnancy born alive?	◯ Yes<br>◯ No<br>◯ Unknown/unsure<br>◯ One baby born alive, one or more born dead
Did the the baby (or babies) from the 15th pregnancy die within the first 30 days (1 month) after birth?	◯ Yes<br>◯ No<br>◯ Unknown/unsure<br>◯ One baby alive, one or more died

What year was the 16th pregnancy?	__________________________
How far along were you when the 16th pregnancy ended?	◯ Before 12 weeks' gestation (early miscarriage)<br>◯ 12-28 weeks' gestation (late miscarriage)<br>◯ 28-32 weeks' gestation (very preterm)<br>◯ 32-36 weeks' gestation (preterm)<br>◯ 37-40 weeks' gestation (term)<br>◯ After 40 weeks' gestation (post-dates)<br>◯ Unknown/unsure
What type of delivery did you have for the 16th pregnancy?	◯ Vaginal (normal)<br>◯ Cesarean
Was the baby (or babies) from the 16th pregnancy born alive?	◯ Yes<br>◯ No<br>◯ Unknown/unsure<br>◯ One baby born alive, one or more born dead
Did the the baby (or babies) from the 16th pregnancy die within the first 30 days (1 month) after birth?	◯ Yes<br>◯ No<br>◯ Unknown/unsure<br>◯ One baby alive, one or more died
What year was the 17th pregnancy?	__________________________
How far along were you when the 17th pregnancy ended?	◯ Before 12 weeks' gestation (early miscarriage)<br>◯ 12-28 weeks' gestation (late miscarriage)<br>◯ 28-32 weeks' gestation (very preterm)<br>◯ 32-36 weeks' gestation (preterm)<br>◯ 37-40 weeks' gestation (term)<br>◯ After 40 weeks' gestation (post-dates)<br>◯ Unknown/unsure
What type of delivery did you have for the 17th pregnancy?	◯ Vaginal (normal)<br>◯ Cesarean
Was the baby (or babies) from the 17th pregnancy born alive?	◯ Yes<br>◯ No<br>◯ Unknown/unsure<br>◯ One baby born alive, one or more born dead
Did the the baby (or babies) from the 17th pregnancy die within the first 30 days (1 month) after birth?	◯ Yes<br>◯ No<br>◯ Unknown/unsure<br>◯ One baby alive, one or more died
What year was the 18th pregnancy?	__________________________
How far along were you when the 18th pregnancy ended?	◯ Before 12 weeks' gestation (early miscarriage)<br>◯ 12-28 weeks' gestation (late miscarriage)<br>◯ 28-32 weeks' gestation (very preterm)<br>◯ 32-36 weeks' gestation (preterm)<br>◯ 37-40 weeks' gestation (term)<br>◯ After 40 weeks' gestation (post-dates)<br>◯ Unknown/unsure

What type of delivery did you have for the 18th pregnancy?	◯ Vaginal (normal)<br>◯ Cesarean
Was the baby (or babies) from the 18th pregnancy born alive?	◯ Yes<br>◯ No<br>◯ Unknown/unsure<br>◯ One baby born alive, one or more born dead
Did the the baby (or babies) from the 18th pregnancy die within the first 30 days (1 month) after birth?	◯ Yes<br>◯ No<br>◯ Unknown/unsure<br>◯ One baby alive, one or more died
What year was the 19th pregnancy?	______________________________
How far along were you when the 19th pregnancy ended?	◯ Before 12 weeks' gestation (early miscarriage)<br>◯ 12-28 weeks' gestation (late miscarriage)<br>◯ 28-32 weeks' gestation (very preterm)<br>◯ 32-36 weeks' gestation (preterm)<br>◯ 37-40 weeks' gestation (term)<br>◯ After 40 weeks' gestation (post-dates)<br>◯ Unknown/unsure
What type of delivery did you have for the 19th pregnancy?	◯ Vaginal (normal)<br>◯ Cesarean
Was the baby (or babies) from the 19th pregnancy born alive?	◯ Yes<br>◯ No<br>◯ Unknown/unsure<br>◯ One baby born alive, one or more born dead
Did the the baby (or babies) from the 19th pregnancy die within the first 30 days (1 month) after birth?	◯ Yes<br>◯ No<br>◯ Unknown/unsure<br>◯ One baby alive, one or more died
What year was the 20th pregnancy?	______________________________
How far along were you when the 20th pregnancy ended?	◯ Before 12 weeks' gestation (early miscarriage)<br>◯ 12-28 weeks' gestation (late miscarriage)<br>◯ 28-32 weeks' gestation (very preterm)<br>◯ 32-36 weeks' gestation (preterm)<br>◯ 37-40 weeks' gestation (term)<br>◯ After 40 weeks' gestation (post-dates)<br>◯ Unknown/unsure
What type of delivery did you have for the 20th pregnancy?	◯ Vaginal (normal)<br>◯ Cesarean
Was the baby (or babies) from the 20th pregnancy born alive?	◯ Yes<br>◯ No<br>◯ Unknown/unsure<br>◯ One baby born alive, one or more born dead

Did the the baby (or babies) from the 20th pregnancy die within the first 30 days (1 month) after birth?	◯ Yes<br>◯ No<br>◯ Unknown/unsure<br>◯ One baby alive, one or more died

SAVE your work!

Was the baby born to you from this current pregnancy admitted to Toto (Paediatric) ward, neonatal intensive care unit (NICU) or ICU (intensive care unit)?	◯ Yes, Toto/Paeds ward<br>◯ Yes, NICU or ICU ward<br>◯ No, the baby stayed with me<br>◯ Unknown/unsure<br>◯ No, baby was not born alive
Did the baby born to you this pregnancy later die?	◯ Yes, the baby died<br>◯ No, baby is still alive<br>◯ No, baby was not born alive<br>◯ Unknown/unsure
When did your baby die?	______________________________
Currently, does the baby born to you this pregnancy have any of the following?<br><br>--> MARK ALL THAT APPLY	☐ Lethargy (sleeping too much and not waking for feeds)<br>☐ Poor feeding<br>☐ Jaundice (yellowing of the eyes or skin)<br>☐ Fever (feels hot)<br>☐ Hypothermia (feels cold)<br>☐ Convulsions<br>☐ Difficult or fast breathing<br>☐ None of the above<br>(*** If any of these features are present, please REFER THE NEONATE for further assessment by a pediatrician ***)

SAVE your work!

Do you know the date of your LAST normal MENSTRUAL period (LNMP)?	◯ Yes<br>◯ No
Before your delivery this time, when was the first day of your last normal menstrual period?	______________________________<br>(Write '01' for the day and/or month, if unknown, and 2900 for the year, if unknown)
Many people go to a clinic while they are pregnant to check on their health and the health of their baby.<br><br>These are called ANTENATAL CARE CLINICS, and there is one at Mbarara Regional Referral Hospital, and there may also be one closer to your home.<br><br>Have you been to an antenatal care clinic (ANC) to see a medical provider during THIS pregnancy?	◯ Yes<br>◯ No

During THIS pregnancy, how many times did you go to antenatal clinic?	◯ 1 time<br>◯ 2 times<br>◯ 3 times<br>◯ 4 times<br>◯ 5 times<br>◯ 6 times<br>◯ 7 times<br>◯ 8 times or more
Have you ever been in the hospital OVERNIGHT as a patient before?<br><br>--> Include any time she was admitted overnight, including for delivery of a baby.	◯ Yes<br>◯ No<br>(Select "Yes" if the client has been in a hospital or health centre overnight before.)
Prior to this admission, when were you last in the hospital overnight as a patient?	◯ Within the last 1 week<br>◯ More than 1 week ago but within the last 1 month<br>◯ Between 1 month and 6 months ago<br>◯ More than 6 months ago but during this pregnancy<br>◯ Prior to this pregnancy<br>(Use the client's best estimate to select the correct answer)
During THIS pregnancy, did you have any of the following routine antenatal tests performed?<br><br>--> Select ALL tests performed at routine ANC visits ONLY	☐ Hemoglobin estimation ("HB" or a test for anemia, "CBC" or complete blood count)<br>☐ Blood smear for malaria parasites (or RDT for malaria)<br>☐ HIV or AIDS test<br>☐ Random blood sugar (blood sugar testing for diabetes)<br>☐ Blood group and/or Rhesus (Rh) testing<br>☐ Ultrasound scan to check on her pregnancy or baby before 28 weeks of amenorrhea<br>☐ Ultrasound scan to check on her pregnancy or baby after 28 weeks of amenorrhea<br>☐ Urinalysis (for infection or hypertension screening for pre-eclampsia)<br>☐ Blood pressure check<br>☐ Hepatitis B testing ("HepB" blood testing)<br>☐ VDRL (Syphilis) test<br>☐ None of the above<br>(Ask to see patient's antenatal care (ANC) card if she does not remember all tests performed)
During THIS pregnancy, were you given any of the following routine antenatal treatments?<br><br>--> Select ALL treatments given, whether they were done at a routine ANC visit or for any other reason	☐ Folic acid<br>☐ Iron tablets (ferrous iron)<br>☐ Combination tablet of iron and folic acid<br>☐ Albendazole or Mebendazole (for de-worming)<br>☐ Fansidar or "SP" for prophylaxis of malaria (EXCLUDING malaria treatment)<br>☐ None of the above<br>(Ask to see patient's antenatal care (ANC) card if she does not remember all treatments)

How many doses of fansidar or SP were you given for malaria prophylaxis, during this pregnancy?	◯ 1<br>◯ 2<br>◯ 3<br>◯ 4<br>◯ 5<br>◯ 6 or more<br>◯ Don't know or can't estimate<br>(*DO NOT include malaria treatment. Include only prophylactic doses.)

Save your work!

Have you EVER taken any medications to treat your HIV infection or to prevent spread of HIV to your baby?<br><br>--> DO NOT INCLUDE SEPTRIN, only ARVs	◯ Yes<br>◯ No<br>◯ Unknown/unsure<br>(Mark "Yes" even if she only took medications for her HIV one time)
Are you currently taking any medications to treat HIV infection (ANTIRETROVIRALS)?<br><br>By currently taking, I mean any medications you have taken in the past 30 days?	◯ Yes<br>◯ No<br>◯ Unknown/unsure<br>(Use this field to record ANTIRETROVIRAL treatment only -- DO NOT record Septrin use here.)
Which medications are you CURRENTLY taking to treat your HIV infection?<br><br>By currently taking, I mean any medications (antiretrovirals) you have taken in the past 30 days?<br><br>--> Select ALL that apply	☐ TLD (TDF/3TC/DTG)<br>☐ Atripla (TDF/FTC/EFV)<br>☐ Triomune (3TC/d4T/NVP)<br>☐ AZT + 3TC + EFV<br>☐ TDF + 3TC + EFV<br>☐ Other (1st other antiretroviral)<br>☐ Other (2nd other antiretroviral)
1st other CURRENT antiretroviral for HIV	__________________________<br>(Write in other antiretrovrial drug taken within the last 30 days)
2nd other CURRENT antiretroviral for HIV	__________________________<br>(Write in other antiretrovrial drug taken within the last 30 days)
Did you start medication(s) to treat your HIV infection before you became pregnant or after you were already pregnant?<br><br>--> DO NOT INCLUDE SEPTRIN, only ARVs	◯ Before I became pregnant<br>◯ After I became pregnant<br>◯ Unknown/unsure
Do you know when you started taking antiretroviral medications to treat your HIV, either the date, month or year you began?	◯ Yes<br>◯ No
When did you first start taking medications to treat your HIV infection? DO NOT INCLUDE SEPTRIN, only ARVs	__________________________<br>(Write '01' for the day and/or month, if unknown, and 2900 for the year, if unknown)

Did you ever CHANGE which medications you were taking to treat your HIV infection?

--> DO NOT INCLUDE SEPTRIN, only ARVs

◯ Yes
◯ No
◯ Unknown/unsure

Which antiretroviral medication were you on before the change?

____
(*Write in the name of the ARV she was taking before she changed treatment. If she doesn't know the name, write a description of the medication.)

Which antiretroviral medication were you on after the change?

____
(*Write in the name of the ARV she was taking after she changed treatment. If she doesn't know the name, write a description of the medication.)

Did you ever STOP medications you were taking to treat your HIV infection?

--> DO NOT INCLUDE SEPTRIN, only ARVs

◯ Yes
◯ No
◯ Unknown/unsure

When did you STOP ARVs?

____
(Write '01' for the day and/or month, if unknown, and 2900 for the year, if unknown)

When did you RE-START ARVs?

____
(Write '01' for the day and/or month, if unknown, and 2900 for the year, if unknown)

Say: "Now I would like to ask you some questions about the way you have been taking (state current ARVs). I need to understand what people are really doing with their medicines. Do not worry about telling me that you don't take all your pills. Some people take all of their medications as they are prescribed while others may not take all of their medications. I need to know what is really happening, not what you think I "want to hear."

Please give me your best guess about what percentage of your ANTIRETROVIRALS (say drug names) you have taken in the last 30 days.

0% means you have taken no ANTIRETROVIRALS (say drug names)

50% means you have taken half your ANTIRETROVIRALS (say drug names)

100% means you have taken every single dose of your ANTIRETROVIRALS (say drug names).

____
(Write in the % ARVs taken in last 30 days (DO NOT USE THE % symbol, ONLY NUMBERS, e.g. 50 for 50%))

Are you currently taking SEPTRIN or BACTRIM (Trimethoprim Sulfamethoxazole or TMP-SMX) to prevent infections?<br><br>By currently taking, I mean any medications you have taken in the past 30 days?	◯ Yes<br>◯ No<br>(Use this field to record SEPTRIN prophylaxis only (and ONLY if the client is taking Septrin to prevent HIV-related infections))
When did you BEGIN taking Septrin or Bactrim? That is, what day did you take the FIRST pill?	______________________<br>(Write "01" for the day or month if the client does know the exact day or month she started Septrin, and 2900 for the year, if not known)
When was your LAST DOSE of Septrin or Bactrim? That is, what day did you take the LAST pill?	______________________<br>(Write "01" for the day or month if the client does know the exact day or month she last took Septrin, and 2900 for the year, if unknown)
Please give me your best guess about how much of your SEPTRIN you have taken in the last 30 days.<br><br>0% means you have taken no SEPTRIN<br><br>50% means you have taken half your SEPTRIN<br><br>100% means you have taken every single dose of your SEPTRIN.	______________________<br>(Write in the % SEPTRIN taken in last 30 days (DO NOT USE THE % symbol, ONLY NUMBERS, e.g. 50 for 50%))
Are you CURRENTLY taking any of these other medications to PREVENT or TREAT INFECTIONS (antibiotics) such as pneumocystis pneumonia or tuberculosis?<br><br>By currently taking, I mean that you've taken the medication in the PAST 30 DAYS.<br><br>--> MARK ALL that apply	☐ Dapsone<br>☐ Atovaquone (Mepron)<br>☐ Isoniazid (INH)<br>☐ Pyrazinamide (PZA)<br>☐ Rifampin (RIF, Rifadin, Rimactane)<br>☐ Ethambutol (EMB, Myambutol)<br>☐ Rifabutin (Mycobutin, Ansamycin)<br>☐ Rifamate (Rifampin/Isoniazid)<br>☐ Rifater (Rifampin/Isoniazid)<br>☐ Other<br>☐ Other<br>☐ NONE of the above
Other CURRENT antibiotic to prevent or treat infection	______________________<br>(Write in other antibiotic taken within the last 30 days)
Other CURRENT antibiotic to prevent or treat infection	______________________<br>(Write in other antibiotic taken within the last 30 days)
Do you think you are currently at risk of acquiring HIV infection	◯ Yes<br>◯ No

Which of the following describe you and your life?<br><br>--> CHECK ALL THAT APPLY	☐ My spouse is HIV-positive<br>☐ My spouse's HIV status is not known to me<br>☐ I have one or more sexual partner(s) who are HIV-positive (Do not include the participant's spouse, if they have one)<br>☐ I have one or more sexual partner(s) whose HIV status is not known to me (Do not include the participant's spouse, if they have one)<br>☐ I work as a sex worker<br>☐ I trade sex for money or gifts<br>☐ I inject drugs<br>☐ I sometimes or always have sex without using a condom<br>☐ I have had more than one sexual partner in the last 1 month<br>☐ I am worried that I might be exposed to HIV another way<br>☐ None of the above
Write other way participant is worried she may be exposed to HIV	__________________________
Are you currently taking any medications to prevent HIV infection?<br><br>By currently taking, I mean any medications you have taken in the past 30 days?<br><br>--> ONLY include antiretroviral medications taken as PrEP	◯ Yes<br>◯ No
Which medications are you currently taking to prevent HIV infection?<br><br>These medications are sometimes called 'PrEP'.<br><br>By currently taking, I mean any medications you have taken in the past 30 days?	◯ Tenofovir disoproxil fumarate (TDF) 300mg / EMTRICITABINE (FTC) 200mg once daily (Truvada)<br>◯ Tenofovir disoproxil fumarate (TDF) 300mg / LAMIVUDINE (3TC) 300mg once daily<br>◯ Tenofovir alafenamide (TAF) 25mg / EMTRICITABINE (FTC) 200mg once daily<br>◯ Other
Write other medication participant is taking to prevent HIV infection<br><br>--> ONLY include antiretroviral medications taken as PrEP	__________________________
Did you start medication to prevent your HIV infection (PrEP) before you became pregnant or after you were already pregnant?<br><br>--> ONLY include PrEP	◯ Before I became pregnant<br>◯ After I became pregnant<br>◯ Unknown/unsure
When did you first start taking medication to prevent HIV infection?<br><br>--> ONLY include PrEP	__________________________<br>(Write '01' for date or month, if not known)
Did you ever STOP taking medication to prevent HIV infection (PrEP)?<br><br>--> ONLY include PrEP	◯ Yes<br>◯ No

When did you STOP medication to prevent HIV infection?<br><br>--> ONLY include PrEP	__________________________________<br>(Write '01' for date or month, if not known)
Why did you stop taking medication (PrEP) to prevent HIV infection?<br><br>--> CHECK ALL THAT APPLY	◯ Side effects from the medication<br>◯ I lost my medication<br>◯ I ran out of medication<br>◯ I stopped while traveling<br>◯ My sexual partner(s) did not want me to take it<br>◯ My family did not want me to take it<br>◯ My PrEP provider (doctor or other clinician) said I should stop<br>◯ Another doctor or clinician (other than my PrEP provider) said I should stop<br>◯ Other
Other reason why you stopped taking PrEP to prevent HIV infection:	__________________________________
After you STOPPED taking medication to prevent HIV infection, did you RESTART medication to prevent HIV infection?<br><br>--> ONLY include PrEP	◯ Yes<br>◯ No
When did you RESTART medication to prevent HIV infection?<br><br>--> ONLY include PrEP	__________________________________<br>(Write '01' for date or month, if not known)
How many times IN A DAY do you typically take your medication (PrEP) to prevent HIV infection?	◯ 0 times (rarely or never)<br>◯ 1 time<br>◯ 1-2 times<br>◯ 2 times<br>◯ More than 2 times a day<br>◯ Only when I have sex without a condom
Did you take your medication (PrEP) to prevent HIV today?	◯ Yes<br>◯ No
Did you take your medication (PrEP) to prevent HIV yesterday?	◯ Yes<br>◯ No
How many times did you take your medication (PrEP) to prevent HIV in the last 7 days?	◯ 0<br>◯ 1<br>◯ 2<br>◯ 3<br>◯ 4<br>◯ 5<br>◯ 6<br>◯ 7<br>◯ More than 7
How well have you taken your PrEP as directed in the past month?	◯ Very poor<br>◯ Poor<br>◯ Fair<br>◯ Good<br>◯ Very good<br>◯ Excellent

In the past month, how often did you take your PrEP?	◯ A little of the time<br>◯ Some of the time<br>◯ A good bit of the time<br>◯ Most of the time<br>◯ All of the time<br>◯ Never
How many doses of PrEP did you miss in the past month?	______________________
Do you think you took PrEP well enough to be protected from HIV?	◯ Yes<br>◯ No<br>◯ Not sure

Now I would like to ask you some questions about the way you have been taking PrEP, the medication you take to prevent HIV infection. I need to understand what people are really doing with their medicines. Do not worry about telling me that you don't take all your pills. Some people take all of their medications as they are prescribed while others may not take all of their medications. I need to know what is really happening, not what you think I "want to hear."

Please give me your best guess about what percentage of your medication to prevent HIV (PrEP) you have taken in the last 30 days.<br><br>0% means you have taken no PrEP<br><br>50% means you have taken half your PrEP<br><br>100% means you have taken every single dose of your PrEP.	______________________<br>(Write in the % PrEP taken in the last 30 days (DO NOT USE THE % symbol, ONLY NUMBERS, e.g. 50 for 50%))
In the last 30 days have you taken/had any of these other medications?<br><br>--> MARK ALL THAT APPLY	☐ Birth control pill (oral hormonal contraceptive)<br>☐ Birth control injection (intramuscular hormonal contraceptive)<br>☐ Birth control implant (implanted hormonal contraceptive)<br>☐ Other<br>☐ No other medications<br>(Write in the % PrEP taken in the last 30 days (DO NOT USE THE % symbol, ONLY NUMBERS, e.g. 50 for 50%))
What other medication did you take in the last 30 days?	______________________<br>(Write in the name(s) and type(s) of any other medication(s))

In the LAST 1 MONTH, has a doctor, midwife or other healthcare provider told you that you have one of these INFECTIONS?<br><br>--> MARK ALL that apply	☐ Malaria<br>☐ Urinary tract infection (UTI)<br>☐ Vaginal infection (including vaginal candiasis, bacterial vaginosis)<br>☐ Sexually transmitted infection (including Chlamydia, Gonorrhea, Herpes, Syphilis)<br>☐ Skin or wound infection<br>☐ Tuberculosis (TB)<br>☐ Other (1st other infection)<br>☐ Other (2nd other infection)<br>☐ NONE of these infections within the last 1 month
1st other infection within the LAST 1 MONTH	______________________________<br>(Write in other infection diagnosed within the last 1 month)
2nd other infection within the LAST 1 MONTH	______________________________<br>(Write in other infection diagnosed within the last 1 month)
PRIOR TO COMING TO HOSPITAL this time, when was the last time a doctor, midwife or other healthcare provider gave you a medication to fight infection (EXCLUDING HIV prophylaxis, such as Bactrim or Septrin)?<br><br>These medications are also called "antibiotics" or "capsules" to treat infection.<br><br>--> MARK ONE	◯ Within the last 1 week<br>◯ More than 1 week ago but within the last 1 month<br>◯ More than 1 month ago but within the last 1 year<br>◯ More than 1 year ago<br>◯ Never

Now I would like to ask you some questions about your health during and before pregnancy, and things that happened while you were here in the hospital. I need to understand what is really happening with your health. Please answer as honestly and completely as you can, not what you think 'I want to hear.' You may find this information sensitive or personal in nature. This information is private and confidential.

Before delivering your baby this time, for how many hours did you have pain in your abdomen that you thought might be from labor (labor-like pains)?	______________________________<br>(in HOURS. Convert all time to hours 30 minutes = 0.5 hours, 60 minutes = 1 hour, 1 day = 24 hours. Record TOTAL amount of time, including time before coming to hospital or health center, and time after coming to the hospital or health center.)
For how many hours did you have labor-like pains BEFORE coming to a hospital or health center?	______________________________<br>(in HOURS. Convert all time to hours 30 minutes = 0.5 hours, 60 minutes = 1 hour, 1 day = 24 hours)

SAVE YOUR WORK!

Can you estimate the number of VAGINAL EXAMS you have had while you were IN LABOR, this pregnancy?	◯ Yes<br>◯ No
What is your best guess of HOW MANY VAGINAL EXAMS you had while you were in labor this time?<br><br>By vaginal exam, I mean where a healthcare provider put their fingers inside your vagina.	__________________________________<br>(Use the client's best estimation of the number of exams)
Before you came to hospital this time, did you have any fever?	◯ Yes<br>◯ No<br>◯ Unknown/unsure
When did your fever begin, to the best of your knowledge?	__________________________________<br>(Write '01' for the day and/or month, if unknown, and 2900 for the year, if unknown)
Have you taken any other medications, tablets or herbal preparations in the last 1 week prior to coming to the hospital this time?	◯ Yes<br>◯ No
What is the name of the medication you took?	__________________________________<br>(*If the participant does not know the name, write a description of what she took)
Have you had any of these other symptoms in the last 1 week?<br><br>--> SELECT ALL THAT APPLY	☐ Chills/feeling cold<br>☐ Shaking chills<br>☐ Abdominal pain<br>☐ Difficulty walking<br>☐ Abnormal vaginal discharge (DO NOT INCLUDE clear vaginal discharge)<br>☐ Foul-smelling vaginal discharge<br>☐ Itching in the vagina or vulva<br>☐ Cough<br>☐ Sputum production<br>☐ Pain with urination or a burning sensation<br>☐ Increased frequency of urination<br>☐ Urinary incontinence (leakage of urine)<br>☐ Headache<br>☐ Other symptom<br>☐ None of the above symptoms last 1 week
Other symptom	__________________________________<br>(List the 'other' symptom the participant has had in the last 1 week)
Have you ever received any vaccination or shot to prevent infection?<br><br>Look in the participant's treatment book for answers to these questions, if she does not know.	◯ Yes<br>◯ No<br>◯ Unsure/does not know
Did you receive any vaccinations or shots to prevent infection as a child?	◯ Yes<br>◯ No<br>◯ Unsure/does not know

Did you receive any vaccinations or shots to prevent infection during pregnancy?	◯ Yes<br>◯ No<br>◯ Unsure/does not know
Do you think you received the measles or MMR vaccine (usually at 9 months of age)?	◯ Yes<br>◯ No<br>◯ Unknown/unsure
In what year (approximately) did you receive the measles or MMR vaccine?	__________________________________<br>(Write 2900 for the year, if not known)
Do you think you received the oral polio vaccine (usually at birth)?	◯ Yes<br>◯ No<br>◯ Unknown/unsure
In what year (approximately) did you receive the oral polio vaccine?	__________________________________<br>(Write 2900 for the year, if not known)
Do you think you received the tetanus toxoid vaccine (usually given during pregnancy)?	◯ Yes<br>◯ No<br>◯ Unknown/unsure
In what year (approximately) did you receive the tetanus toxoid vaccine?	__________________________________<br>(Write 2900 for the year, if not known)
Do you think you received the BCG vaccine (usually at birth)?	◯ Yes<br>◯ No<br>◯ Unknown/unsure
In what year (approximately) did you receive the BCG vaccine?	__________________________________<br>(Write 2900 for the year, if not known)
Do you think you received the DPT-Hep-Hib vaccine (usually at 6 weeks of age)?	◯ Yes<br>◯ No<br>◯ Unknown/unsure
In what year (approximately) did you receive the DPT-Hep-Hib vaccine?	__________________________________<br>(Write 2900 for the year, if not known)
Do you think you received the PCV vaccine (usually at 6 weeks of age)?	◯ Yes<br>◯ No<br>◯ Unknown/unsure
In what year (approximately) did you receive the PCV vaccine?	__________________________________<br>(Write 2900 for the year, if not known)
Do you think you received the HPV vaccine (usually at 10 years of age)?	◯ Yes<br>◯ No<br>◯ Unknown/unsure

In what year (approximately) did you receive the HPV vaccine?	______________________ (Write 2900 for the year, if not known)
Do you think you received the IPV (polio injection) vaccine (usually at 14 weeks of age)?	◯ Yes ◯ No ◯ Unknown/unsure
In what year (approximately) did you receive the IPV (polio injection) vaccine?	______________________ (Write 2900 for the year, if not known)
Do you think you received the rota vaccine (usually at 6 weeks of age)?	◯ Yes ◯ No ◯ Unknown/unsure
In what year (approximately) did you receive the rota vaccine?	______________________ (Write 2900 for the year, if not known)
Do you think you received the any other vaccine?	◯ Yes ◯ No ◯ Unknown/unsure
In what year (approximately) did you receive the other vaccine?	______________________ (Write 2900 for the year, if not known)
RA doing pain assessment at delivery<br><br>HIDE after this until...	◯ NP ◯ AK ◯ MaS ◯ NM ◯ NR ◯ AB ◯ TR ◯ AS ◯ MoS ◯ MA ◯ EA ◯ ET ◯ AA ◯ DM ◯ AB ◯ FTA
What was the pain assessment at delivery	◯ 0 ◯ 1 ◯ 2 ◯ 3 ◯ 4 ◯ 5 ◯ 6 ◯ 7 ◯ 8 ◯ 9 ◯ 10
RA doing acceptability (QUIC and medical research) questionnaire	◯ NP ◯ AK ◯ MaS ◯ NM ◯ NR ◯ AB ◯ TR ◯ AS ◯ MoS ◯ MA ◯ EA ◯ ET ◯ AA ◯ DM ◯ AB ◯ FTA
post-PACO (QUIC) Assessments	
Date post-PACO (QUIC) assessment performed	______________________

RA doing post-PACO (QUIC) assessment	◯ NP ◯ AK ◯ MaS<br>◯ NM ◯ NR ◯ AB<br>◯ TR ◯ AS ◯ MoS<br>◯ MA ◯ EA ◯ ET ◯ AA<br>◯ DM ◯ AB ◯ FTA
What is your current pain level?	◯ 0 ◯ 1 ◯ 2 ◯ 3<br>◯ 4 ◯ 5 ◯ 6 ◯ 7<br>◯ 8 ◯ 9 ◯ 10
Please enter the findings of the Medical Research Trust Scale	
Doctors who do medical research care only about what is best for each patient.	◯ Strongly disgree<br>◯ Disagree<br>◯ Neither agree or disagree<br>◯ Agree<br>◯ Strongly Agree
Doctors tell their patients everything they need to know about being in a research study.	◯ Strongly disgree<br>◯ Disagree<br>◯ Neither agree or disagree<br>◯ Agree<br>◯ Strongly Agree
Medical researchers treat people like "guinea pigs"	◯ Strongly disgree<br>◯ Disagree<br>◯ Neither agree or disagree<br>◯ Agree<br>◯ Strongly Agree
I completely trust doctors who do medical research	◯ Strongly disgree<br>◯ Disagree<br>◯ Neither agree or disagree<br>◯ Agree<br>◯ Strongly Agree
Please enter the findings of the State Trait Anxiety Scale	
I feel calm	◯ Not at all<br>◯ Somewhat<br>◯ Moderately so<br>◯ Very much so
I feel tense	◯ Not at all<br>◯ Somewhat<br>◯ Moderately so<br>◯ Very much so
I feel upset	◯ Not at all<br>◯ Somewhat<br>◯ Moderately so<br>◯ Very much so
I feel relaxed	◯ Not at all<br>◯ Somewhat<br>◯ Moderately so<br>◯ Very much so

I feel content	◯ Not at all<br>◯ Somewhat<br>◯ Moderately so<br>◯ Very much so
I feel worried	◯ Not at all<br>◯ Somewhat<br>◯ Moderately so<br>◯ Very much so
Please enter the findings of the QUIC Part A Scale	
When I signed the consent form for PACO, I knew I was agreeing to participate in a clinical research study.	◯ Disagree<br>◯ Unsure<br>◯ Agree
The main reason clinical research studies are done is to improve the treatment of future patients	◯ Disagree<br>◯ Unsure<br>◯ Agree
I was informed how long I would participate in this research study	◯ Disagree<br>◯ Unsure<br>◯ Agree
The monitoring I received is standard for women who have undergone delivery	◯ Disagree<br>◯ Unsure<br>◯ Agree
In my study, one of the researchers' major purposes is to understand changes in the placenta that can affect a woman's baby	◯ Disagree<br>◯ Unsure<br>◯ Agree
In my clinical research study, one of the researchers' major purposes is to find out how changes in the placenta affect a woman's baby.	◯ Disagree<br>◯ Unsure<br>◯ Agree
The questions being researched in my clinical research study have already been answered in other studies	◯ Disagree<br>◯ Unsure<br>◯ Agree
Compared with standard ways of monitoring and treating women who are delivering their baby, my clinical research study does not carry any additional risks or discomforts	◯ Disagree<br>◯ Unsure<br>◯ Agree
There may not be direct medical benefit to me from my participation in this clinical research study.	◯ Disagree<br>◯ Unsure<br>◯ Agree
By participating in this clinical research study, I am helping the researchers learn information that may benefit other women delivering their babies.	◯ Disagree<br>◯ Unsure<br>◯ Agree
Because I am participating in a clinical research study it is possible that the study sponsor, various government agencies or others who are not directly involved in my care could review my medical records.	◯ Disagree<br>◯ Unsure<br>◯ Agree

My doctors did not offer me any alternatives besides participating in this clinical research study	◯ Disagree<br>◯ Unsure<br>◯ Agree
The consent form I signed lists the name of the person (or persons) whom I should contact if I have any questions or concerns about the clinical research study	◯ Disagree<br>◯ Unsure<br>◯ Agree
If I had not wanted to participate in this clinical research study, I could have declined to sign the consent form.	◯ Disagree<br>◯ Unsure<br>◯ Agree
I had to remain in the clinical research study even if I decided I wanted to withdraw.	◯ Disagree<br>◯ Unsure<br>◯ Agree
Please enter the findings of the QUIC Part B Scale	
The fact that the measurements and samples you had involves research	◯ Did not understand at all<br>◯ Understood very little<br>◯ Understood somewhat<br>◯ Understood most<br>◯ Understood completely
What the researchers are trying to find out in the clinical research study	◯ Did not understand at all<br>◯ Understood very little<br>◯ Understood somewhat<br>◯ Understood most<br>◯ Understood completely
How long you would be in the clinical research study	◯ Did not understand at all<br>◯ Understood very little<br>◯ Understood somewhat<br>◯ Understood most<br>◯ Understood completely
The procedures you would undergo	◯ Did not understand at all<br>◯ Understood very little<br>◯ Understood somewhat<br>◯ Understood most<br>◯ Understood completely
Which of these procedures are experimental	◯ Did not understand at all<br>◯ Understood very little<br>◯ Understood somewhat<br>◯ Understood most<br>◯ Understood completely
The possible risks and discomforts of participating in the clinical research study	◯ Did not understand at all<br>◯ Understood very little<br>◯ Understood somewhat<br>◯ Understood most<br>◯ Understood completely

The possible benefits to you of participating in the clinical research study	◯ Did not understand at all<br>◯ Understood very little<br>◯ Understood somewhat<br>◯ Understood most<br>◯ Understood completely
How your participation in this clinical research study may benefit future patients	◯ Did not understand at all<br>◯ Understood very little<br>◯ Understood somewhat<br>◯ Understood most<br>◯ Understood completely
The alternatives to participation in the clinical research study	◯ Did not understand at all<br>◯ Understood very little<br>◯ Understood somewhat<br>◯ Understood most<br>◯ Understood completely
The effect of the clinical research study on the confidentiality of your medical records	◯ Did not understand at all<br>◯ Understood very little<br>◯ Understood somewhat<br>◯ Understood most<br>◯ Understood completely
Whom you should contact if you have questions or concerns about the clinical research study	◯ Did not understand at all<br>◯ Understood very little<br>◯ Understood somewhat<br>◯ Understood most<br>◯ Understood completely
The fact that the participation in the clinical research study is voluntary	◯ Did not understand at all<br>◯ Understood very little<br>◯ Understood somewhat<br>◯ Understood most<br>◯ Understood completely
Overall, how well did you understand your clinical research study when you signed the consent form	◯ Did not understand at all<br>◯ Understood very little<br>◯ Understood somewhat<br>◯ Understood most<br>◯ Understood completely
